# Material Classification from Non-Line-of-Sight Acoustic Echoes Using Wavelet-Acoustic Hybrid Feature Fusion

**DOI:** 10.3390/s26051577

**Published:** 2026-03-03

**Authors:** Dilan Onat Alakuş, İbrahim Türkoğlu

**Affiliations:** 1 Department of Software Engineering, Faculty of Engineering, Kırklareli University, Kırklareli 39100, Türkiye; 2 Department of Software Engineering, Faculty of Technology, Fırat University, Elazığ 23119, Türkiye; iturkoglu@firat.edu.tr

**Keywords:** NLOS acoustic sensing, wavelet feature fusion, hybrid acoustic features, deep recurrent networks

## Abstract

**Highlights:**

**What are the main findings?**

- The proposed Wavelet–Hybrid CNN–LSTM model achieved 99% balanced accuracy and macro-F1 score in classifying materials from non-line-of-sight (NLOS) acoustic echoes, outperforming wavelet-only and classical acoustic models.
- SHAP-based explainability analysis revealed that Mel-Frequency Cepstral Coefficient (MFCC) and wavelet entropy-energy features play complementary roles in material discrimination, allowing the model to not only classify with high accuracy but also interpret physical material properties such as hardness, density, and porosity.

**What are the implications of the main findings?**

- The study demonstrates that hybrid wavelet–acoustic feature fusion can enable real-time, interpretable acoustic sensing systems for material recognition in NLOS environments such as robotics, defense, and industrial monitoring.
- The integration of deep recurrent models with interpretable hybrid features provides a foundation for developing physics-informed artificial intelligence systems, bridging the gap between data-driven learning and acoustic material physics.

**Abstract:**

Acoustic material classification under non-line-of-sight (NLOS) conditions—where direct sound paths are obstructed—is a challenging task due to echo attenuation, complex reflections, and noise effects. This study aims to improve NLOS material recognition by introducing a novel wavelet–acoustic hybrid feature fusion method integrated with deep recurrent neural network architectures. Echo signals from nine different materials were collected using the newly developed ANLOS-R (Acoustic Non-Line-of-Sight Recognition) dataset, which was specifically designed to simulate realistic NLOS propagation environments. From these recordings, time-domain acoustic features and multi-scale wavelet-based energy and entropy statistics were extracted using ten wavelet families. The resulting 70-dimensional hybrid feature set was used to train several deep learning architectures, including Long Short-Term Memory (LSTM), Bidirectional LSTM (BiLSTM), Gated Recurrent Unit (GRU), and Convolutional Neural Network–LSTM (CNN–LSTM). Among these, the CNN–LSTM achieved the highest balanced accuracy and macro-F1 score of 0.99, showing strong generalization and convergence performance. SHapley Additive exPlanations (SHAP) analysis indicated that Mel-Frequency Cepstral Coefficients (MFCCs) and wavelet entropy–energy features play complementary roles in material discrimination. The proposed approach provides a robust and interpretable framework for real-time NLOS acoustic sensing, bridging data-driven deep learning with the physical understanding of acoustic material behavior.

## 1. Introduction

In recent years, acoustic signal processing has gained increasing importance, particularly for material identification and environmental perception under non-line-of-sight (NLOS) conditions. NLOS acoustic sensing refers to scenarios in which the direct line of sight is obstructed, and sound waves reach the target by reflecting off one or more surfaces. In such environments, the analysis of echo signals plays a critical role in determining the physical properties of the environment and the surface materials [1]. However, signal echo attenuation, noise effects, and complex reflection patterns in NLOS conditions complicate acoustic signal analysis and reduce classification accuracy [2]. Therefore, the accurate modeling of echo signals and the extraction of information-bearing features have become a fundamental research area in NLOS-based acoustic sensing.

Particularly in ultra-wideband (UWB) and acoustic-based positioning systems, the detection and classification of NLOS conditions directly affect system accuracy [3]. The frequency, amplitude, and temporal components of acoustic echoes provide important information about the physical structure of the material from which the sound is reflected. These aspects of echo signals are evaluated in many applications, such as industrial material identification, security, defense, and health. Wavelet-based analysis methods achieve high performance in noise reduction and feature extraction by decomposing acoustic signals in the time-frequency domain [4]. Studies in the literature have indicated that wavelet transformation yields superior results compared to traditional Fourier methods in the analysis of non-stationary acoustic signals [5,6,7]. Furthermore, it has been demonstrated that wavelet-based features can be effectively used to increase the signal-to-noise ratio of echo signals and enhance the discrimination of target materials [8]. For instance, the improved wavelet denoising method developed by Tian Gao and colleagues increased the Signal/Noise Ratio (SNR) value of the echo signal, enabling more accurate determination of the target position [9]. Wavelet-based features are typically used in a hybrid manner alongside classical acoustic parameters such as Mel-Frequency Cepstral Coefficient (MFCC), Root Mean Square (RMS), Zero Crossing Rate (ZCR), spectral centroid, and bandwidth. These hybrid approaches enhance classification performance by representing both the spectral and temporal characteristics of the signal [10]. Higher accuracy rates have been achieved by combining wavelet-based spectral features with MFCC features in the classification of bird and fish sounds [11,12]. Similarly, various studies have reported that models developed using wavelet–MFCC combinations yield more stable and discriminative results compared to MFCC-based approaches alone [11]. In recent years, deep learning-based models have made significant progress in the classification of acoustic signals. Recurrent neural networks, particularly Long Short-Term Me-mory (LSTM) and Gated Recurrent Unit (GRU), have been successful in learning long-term dependencies in time series. The literature reports that combining wavelet scattering-based features with GRU networks achieves higher accuracy in acoustic environment classification compared to LSTM-based methods [13]. These models demonstrate strong generalization capabilities even under NLOS conditions by capturing the temporal patterns of echo signals.

In this study, unlike existing approaches in the literature, it is proposed to combine features extracted using different wavelet functions (coif1, bior3.3, db2, etc.) with classical acoustic features in a hybrid manner and to classify these features using deep recurrent models such as LSTM, Bidirectional LSTM (BiLSTM), GRU, and Bidirectional GRU (BiGRU) and hybrid Convolutional Neural Network-LSTM (CNN-LSTM). The study also ranks feature importance using multiple statistical analyses and the SHapley Additive exPlanations (SHAP) method, thereby increasing the explainability of the model’s decision mechanism. In the study by Li et al. [3], which used SHAP analysis for classifying NLOS signals, it was noted that even the four most important features provided high accuracy. Such explainability-based analyses are particularly important in acoustic-based deep learning systems to understand which features the model relies on. The proposed wavelet–hybrid feature fusion and deep recurrent model-based method in this study offer an innovative approach to improve material classification accuracy from NLOS echo signals. The joint evaluation of both the time-frequency representations and the statistical significance of the features contribute meaningfully to the literature in the fields of acoustic signal processing and material classification. In conclusion, the methods used in this study provide a novel framework for estimating material types from echo-based acoustic signals. The proposed Wavelet–Hybrid CNN–LSTM architecture demonstrated high balanced accuracy and physical consistency under NLOS echo conditions. In this study, the combination of multi-scale features derived from ten different wavelet families and classical acoustic parameters has made the material’s acoustic reflection, absorption, and scattering properties interpretable by the deep learning model. The results obtained reveal that both wavelet-based features and the time-frequency hybrid representation create significant synergy in echo-based acoustic material classification. The main contributions of this study are summarized below:

- The ANLOS-R dataset was generated for the study. Echo signals from different material types were recorded and each sample file was associated with a label.
- A new feature extraction approach based on the combination of wavelet and classical acoustic (hybrid) features was developed. Multi-level statistical features (energy, entropy, skewness, kurtosis, etc.) extracted using coif1, bior3.3, and db2 wavelet functions were combined with acoustic parameters such as MFCC, RMS, and ZCR.
- Deep learning models based on LSTM, BiLSTM, GRU, and BiGRU were trained and compared on the hybrid feature set. These models achieved high accuracy in NLOS material classification by learning the temporal dependencies of echo signals.
- An explainability assessment based on SHAP was performed. This determined which features played a dominant role in the model’s decision mechanism and applied explainable artificial intelligence principles.
- The contribution rates of wavelet and hybrid feature groups in classification were examined separately, and the overall impact levels of wavelet families (coif1, bior3.3, db2) were quantitatively evaluated.

The remaining sections of this article are organized as follows: In Section 2, the literature on NLOS acoustic sensing, material recognition, and wavelet-based sound processing is comprehensively reviewed, and the limitations of existing methods are discussed. Section 3 is devoted to the dataset used in the study and the feature extraction process; detailing the nine-class acoustic dataset created from NLOS echo signals, the extraction of hybrid acoustic features such as RMS, ZCR, and MFCC, the calculation of multi-level wavelet features generated using coif1, bior3.3, and db2 wavelet functions, and the feature fusion process combining these two groups of features. Furthermore, this section covers the structure of deep recurrent models, their training parameters and evaluation metrics. Section 4 provides the experimental findings with including balanced accuracy, F1-macro and gain score. Furthermore, this section focuses experimental results in detail and provide visualizations including Receiver Operating Characteristic (ROC) curves, confusion matrices, loss and accuracy graphs related to the training process, and SHAP-based feature importance rankings are presented for each model. In addition, the relative contribution rates of wavelet and hybrid feature groups in classification are examined. Section 5 evaluates the obtained results; the advantages of the wavelet–hybrid combination over classical MFCC-based models and the physical counterparts of specific wavelet levels and model interpretability based on SHAP analysis are evaluated. In Section 6, the general findings of the study are summarized, the success of NLOS echo-based material recognition enhanced by wavelet–hybrid synergy is emphasized, and suggestions for future research are presented.

## 2. Related Works

In recent years, NLOS acoustic sensing has gained increasing importance, particularly in areas such as indoor positioning, industrial monitoring, autonomous systems, and acoustic material classification. Much of the work in this field focuses on determining material types or object locations by analyzing the temporal and frequency-related characteristics of echo signals. Traditional methods typically rely on manual feature extraction or threshold-based analysis techniques, which are noise-sensitive and limited in terms of generalizability. Therefore, deep learning-based automatic feature extraction approaches have emerged in recent years to provide higher accuracy and robust-ness in NLOS acoustic scenes. In this context, one of the prominent models in the literature, the Channel Attention Mechanism-LSTM (CAM–LSTM) based 1D-CNN architecture, emphasizes important acoustic features using the CAM while learning the temporal relationships of echo signals through the LSTM layer. Studies using this method have achieved accuracy levels of 97–98% in tests conducted on different environmental data, demonstrating that the model’s overall performance is superior to traditional classifiers [14].

On the other hand, wavelet-based feature extraction enables a multiscale time-frequency representation of acoustic signals, allowing for better separation of echo and resonance components. Genetic algorithm-optimized wavelet transform approaches have significantly reduced error rates in both regression and classification tasks by selecting meaningful peaks in the resonance spectrum that are free from noise. Improvements of up to 95% in regression error and 40% in classification error have been reported [15]. In addition, the hybrid feature extraction approach—combining features obtained from deep learning models with classical statistical and frequency-based features is widely used in acoustic signal classification. Deep features extracted from pre-trained models such as NASNetLarge and NASNetMobile were selected using the chi^2^ method and fed into Support Vector Machine (SVM), resulting in 77.66% accuracy. This result demonstrates that hybrid feature fusion provides a significant performance increase compared to the use of single models [16]. In echo-based material classification, image-based approaches have been integrated with acoustic modeling. Surface patches extracted from images are fed into the classifier to estimate frequency-dependent acoustic absorption coefficients, and these values are used in the production of the Room Impulse Response (RIR). The results obtained show that RIRs produced by automatic labeling are indistinguishable from manually labeled RIRs, both objectively and subjectively [17].

Approaches combining wavelet-based time-frequency analysis with deep learning have also gained significant attention in the literature. Processing acoustic images obtained with Continuous Wavelet Transform (CWT) using Convolutional Autoencoder (CAE) and CNN has enabled the extraction of both local and global features. This structure has ensured high accuracy and low error rates in pipe-line leak detection [18]. Furthermore, the combination of wavelet + genetic algorithm has been shown to create noise-insensitive features with preserved physical meaning in resonance spectrum analysis [19]. Finally, deep learning-based automatic feature extraction is an effective method for learning meaningful representations directly from the spectra of acoustic signals. Features extracted from Fast Fourier Transform (FFT) graphs of acoustic emission signals using CNN-based models, when used in conjunction with t-SNE and Principal Component Analyses (PCA), have achieved high accuracy in wear and fault detection [20]. Furthermore, weighted multi-scale CNN architectures with CAM have stood out in anti-noise experiments with high accuracy and low computation time [21].

Table 1 summarizes the methodological differences between the main NLOS acoustic classification studies in the literature and the contributions of this study. As seen, while most existing studies are limited to tasks such as LOS/NLOS discrimination or position estimation, complex problems such as echo region isolation and material classification have mostly been neglected. In this study, however, the direct classification of material types from echo signals is targeted using wavelet–hybrid feature fusion and LSTM/GRU-based deep network architectures. Thus, a comprehensive NLOS acoustic recognition approach integrating echo isolation, acoustic feature analysis, and deep learning-based classification is presented. As seen in Table 1, most existing studies are limited to classifying NLOS signals or estimating their location; complex tasks such as echo region isolation and material type identification are generally not addressed. This study fills this gap with both wavelet-acoustic feature fusion and SHAP-supported explainable deep learning models.

The vast majority of existing studies focus on problems such as NLOS/LOS separation, fault detection, resonance analysis, or stage reconstruction, and do not directly address surface material type classification under passive acoustic excitation and strict NLOS propagation conditions. In this context, the proposed study addresses a unique problem definition, not directly addressed in the literature, using reverberation region isolation, wavelet-based feature extraction, and a CNN-LSTM-based deep learning architecture.

## 3. Methodology

**Figure 1:**
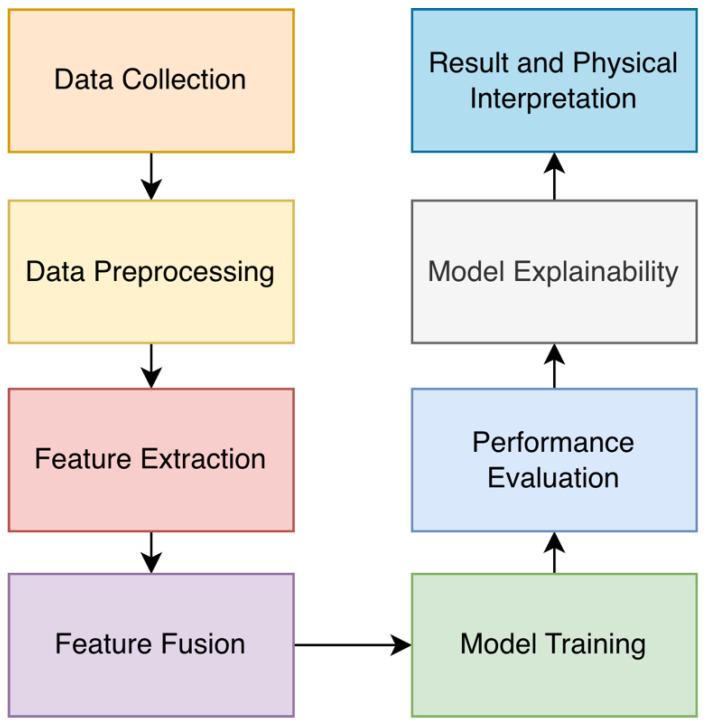
Workflow of the study.

This section presents the general framework of the hybrid deep learning-based method designed to perform material classification from NLOS echo signals. The proposed approach aims to create a multidimensional feature set consisting of both classical acoustic features and time–frequency-based features obtained through wavelet transformation. This hybrid feature set is trained using time series-focused deep learning models such as LSTM, BiLSTM, GRU, BiGRU, GRU-Attention, and BiGRU-Attention, and the model with the highest classification performance is selected for final evaluation. The general flow of this study is summarized in the system diagram presented in Figure 1.

When examining Figure 1, in the first stage, echo recordings from nine different materials were obtained using the ANLOS-R dataset. Subsequently, echo regions (0.65–19.2 ms) were identified on the raw signals through preprocessing. Then, both time–frequency domain-based acoustic features and multi-scale wavelet features obtained from different wavelet families were extracted from the signals. Each .wav audio recording is represented by a single 70-dimensional feature vector using wavelet-based and statistical feature extraction methods. No time windowing or segment-based splitting was applied to the recordings. Therefore, each row in the dataset represents an independent and complete audio recording. After feature extraction, the resulting feature matrix was split into two subsets using random sampling at the recording level, with 80% for training and 20% for testing. Normalization was performed after the data splitting step. The normalization parameters (Z-score) were calculated using only the training data set, and the same parameters were applied to the test data set. This approach prevented any information transfer from the test data to the training process and ensured complete independence between the training and test sets. Since feature extraction is a deterministic transformation that does not use class labels, it does not cause information leakage between examples. The obtained features were trained on different deep learning models for echo-based material classification. After training, the model’s performance was evaluated using balanced accuracy, F1-macro, and ROC-AUC (ROC-Area Under Curve) metrics. In the final stage, the model’s decision process was examined using SHAP-based interpretability analysis, and the effect of features on material types was interpreted.

### 3.1. Dataset and Feature Extraction

**Figure 2:**
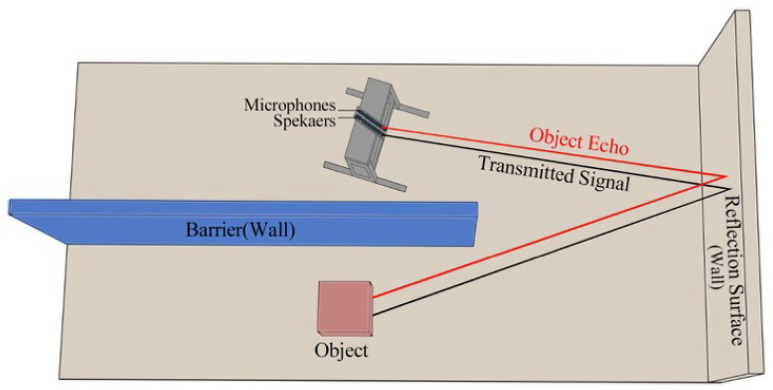
Experimental setup to collect signals.

The ANLOS-R dataset used in this study was specifically created for echo-based material classification under NLOS acoustic conditions, where the direct LOS is obstructed. Figure 2 shows the experimental setup and the methods used for the study.

Figure 2 shows that the data acquisition system consists of a multi-channel setup containing eight speakers and eight microphones positioned parallel to the wall. The speakers and microphones are placed facing the wall; the target material objects are positioned in the same room in such a way that the direct line of sight is obstructed. Measurements were performed at three different positions: reference (0 cm), when the system was shifted +5 cm to the right and −5 cm to the left. This multi-positioning allowed different reflection angles to be recorded and increased the position-independent generalizability of the data set. Measurements were repeated in single speaker–microphone pairs and multiple input/multiple output (MIMO) configurations; each scenario was repeated three times. A total of 1440 audio samples (1152 single, 144 multiples, and 144 background measurements) were collected.

### 3.2. NLOS Acoustic Dataset

The dataset contains nine basic material classes: wood, white goods surface, glass, iron, foam, marble, PVC, sheet metal, and brick. Additionally, realistic reverberation conditions were created by including mixed combinations (e.g., “marble–brick–wood”). Each measurement is recorded in .wav format, and file naming (e.g., NLB3-0_T1_1.wav) is standardized to encode the material type, measurement position, recording type, and repetition number. The chirp (linearly frequency-modulated) signal emitted by the speaker in the 0–20 kHz range reflects off the wall surface before reaching the target directly; this reflection reaches the microphone after hitting the target object. Thus, only the indirect echo (NLOS echo) components are recorded, while the direct signal components are blocked by acoustic isolation. This structure forms the physical basis for the echo-based material classification approach of the study. Only single material classification was performed in this study, and the dataset content is shown in Table 2.

This study aims not only to classify different material types but also to determine the characteristic acoustic properties of each material. To this end, SHAP-based interpretability analysis was applied to examine the impact of the features used in the training phase on model decisions. The analysis process is aimed at evaluating the effects of MFCC, RMS, ZCR, spectral, and wavelet-based energy–entropy features extracted from NLOS echo signals on different material classes. In this context, the weight coefficients assigned by the SHAP method to each feature of the model were calculated, and material-based feature importance profiles were obtained. This approach aims not only to increase the classification accuracy of echo signals but also to statistically explain which features represent the unique acoustic behavior of each material. Therefore, the analyses presented in the subsequent stages of the methodology are structured to reveal the material–acoustic property interaction through SHAP-based evaluations.

### 3.3. Hybrid Acoustic Features

The hybrid acoustic features used in this study were created to comprehensively represent both the energy-based and spectral-frequency-based characteristics of echo signals. Twenty-four basic acoustic features were extracted for each sound sample. These features include the signal’s temporal energy, frequency distribution, statistical structure, and MFCC components. Among the energy-based features, RMS represents the average energy content and variance of the echo signal [22]. The RMS value is directly related to the amplitude intensity of the echo and reflects the hardness and absorption properties of the material [23]. The entropy value represents the degree of irregularity and richness of the echo, while kurtosis and skewness define the shape of the statistical distribution, revealing the symmetry or asymmetry of the echo [24].

Spectral-based features include ZCR, spectral centroid (SC), spectral bandwidth (SB), spectral rolloff (SR), spectral flatness (SF) [25]. ZCR measures the frequency density of the signal and the presence of high-frequency components, while the centroid indicates the center of gravity of the energy density on the frequency axis [26]. Bandwidth and rolloff indicate the signal’s spectrum width and energy distribution, while the flatness value defines the tonal or noisy character of the echo [27]. Additionally, 13 MFCC were calculated to model echo signals in a manner like the human auditory system. The MFCCs represent the formant structure and resonance characteristics of the acoustic energy reflected from the material surface, playing a particularly important role in determining the differences between the reverberation time and the surface acoustic impedance. Average values were taken for each MFCC, thus obtaining a statistical summary of the frequency-based acoustic structure. As a result, the hybrid feature set consists of 24 features that include both the energy-temporal and frequency-resonance dimensions of the echo signals. This structure provides a robust foundation for enabling material classification from echo signals and, when combined with wavelet-based time–frequency features in subsequent stages, creates a richer feature space.

### 3.4. Wavelet Feature Extraction

**Figure 3:**
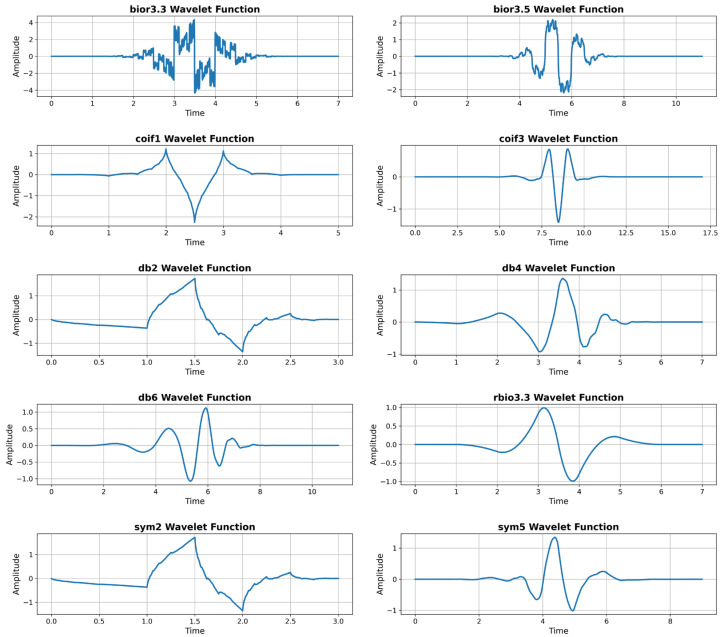
Mother functions of the wavelet transformations.

In this study, 10 different wavelet families were used to decompose the multiscale components of echo signals in the time–frequency domain: bior3.3, bior3.5, coif1, coif3, db2, db4, db6, rbio3.3, sym2, and sym5. Each wavelet function represents the short-term energy distribution and frequency components of echo signals at different resolution levels. Wavelet transformation is particularly suitable for analyzing non-stationary (time-dependent) acoustic signals such as echo signals, providing higher separation power compared to classical Fourier representation [28]. Figure 3 shows the mother functions (ψ(t)) of the wavelet transformations used.

Each wavelet family offers different time–frequency resolution characteristics while representing a specific frequency range of the signal. For instance, the bior and sym families provide stability against deformation with their symmetric structures; the db and coif families exhibit sensitivity to high-frequency components due to their short support lengths. Therefore, the selected wavelet families have been determined to provide balance in both low and high frequency regions.

Recent studies show that time-frequency-wavelet fusion provides a significant performance improvement over classical Fourier-based or purely spectral analysis approaches in acoustic scene classification and non-stationary signal analysis [29,30]. In this context, the 10 wavelet families we selected were chosen because they have the capacity to capture the time, frequency, and transient components of NLOS echo signals.

### 3.5. Time-Frequency Decomposition

For each sound sample, the echo region s was selected, and DWT (Discrete Wavelet Transform) was applied to this signal. The mathematical DWT representation of a signal is given in Equation (1):

(1) $$x\left(t\right)=\sum_{j=0}^{J-1}\sum_{k}c_{j,k}\psi_{j,k}(t)$$

where $\psi_{j,k}\left(t\right)=2^{-\frac{j}{2}}\psi (2^{-j}t-k)$ are scaled and shifted wavelets; $c_{j,k}$ represents the wavelet coefficients. The resolution level is automatically determined for each signal. In this process, the maximum level $J_{max}$ is calculated according to the following relationship given in Equation (2):

(2) $$J_{max}=\log_{2}\left(\frac{N}{L}\right)$$

where N is the signal length, L refers to the wavelet filter length. The average decomposition level varied between 7 and 9, so each signal was analyzed at the resolution most suitable for its length.

### 3.6. Coefficient-Based Statistical Features

Six fundamental statistical features were calculated based on the coefficient sequences $\left(c_{j}\right)$ obtained from each wavelet decomposition and are presented in Table 3. These features were calculated separately for each decomposition level $L_{j}$ and are numbered as $L_{1},L_{2},\ldots ,\;L_{6},\;L_{7}$.

Subsequently, these wavelet-based features were combined with the acoustic feature set via the “File” identifier, and all values were scaled using z-score normalization. This combined feature vector (hybrid set) integrates both low-level statistical information (e.g., RMS, ZCR) and high-level time–frequency features (e.g., wavelet entropy, energy_L3) within the same structure. This allows the model to simultaneously learn the global energy trends and local resonance patterns of the echo signal. An important point to note is that although the skewness and kurtosis values common to hybrid and wavelet features are similar in terms of statistical formulation, the data spaces in which they are applied are different. On the acoustic side, these measures are calculated directly on the wave amplitude or spectral density distribution, while on the wavelet-based side, they represent the statistical distribution of coefficients at each frequency scale (e.g., L3, L4). This difference allows the model to learn both the energy asymmetry in the time domain and the intensity and resonance patterns in the frequency scales.

### 3.7. Feature Fusion

In this study, hybrid acoustic features extracted from echo signals were integrated with wavelet-based time–frequency features to create a single extended feature vector. A total of 70-dimensional feature set was obtained. This set combines the amplitude-based, spectral, statistical, and multi-scale components of the signal within the same data structure. This multi-faceted representation is designed to encompass not only the average energy levels of the echo signals but also the frequency density changes occurring over time and the variations in the echo envelope. This approach enables the deep network to learn both low-level and high-level representations simultaneously by utilizing all the information in different representation spaces (time-domain and wave-let-domain) of the signal. Table 4 shows the total number of features.

Table 4 summarizes the source domains, dimensions, and content types of the feature sets used in the study. Acoustic features consist of spectral summaries based on RMS, ZCR, and MFCC; while wavelet components include energy and entropy statistics obtained from different wavelet families. The fusion set is created by combining these two domains and is normalized using the Z-score method.

### 3.8. Deep Learning Models

In this study, considering the time series nature of acoustic and wavelet-based features obtained from echo signals, various deep learning architectures suitable for sequential data modeling were evaluated. Each of the models used provides different levels of temporal dependency, contextual awareness, and representational power.

The LSTM model was used to learn sequential patterns and long-term dependency relationships in echo signals. On the other hand, the BiLSTM model represented bidirectional temporal dependencies by processing both forward and backward flows of the time series. GRU was implemented as a lightweight recurrent neural network architecture that uses a gate mechanism similar to the LSTM structure but with fewer parameters. BiGRU is the bidirectional version of GRU and processes the signal in both past and future contexts. Temporal attention layers were added to emphasize the relative importance of features at specific time steps. In this context, GRU-Attention, BiGRU-Attention, and BiLSTM-Attention models were created by adding an attention module to the basic recurrent structures.

Finally, the CNN-LSTM architecture is designed as a structure that integrates local features obtained through convolutional layers with LSTM layers in the temporal plane. Both the wavelet-only and hybrid (wavelet + acoustic) feature sets were trained separately using the same model architectures, training parameters, and early stopping strategy. Table 5 shows the architectural structures and training parameters of the deep learning models used in the study.

All models were trained using the Adam optimization algorithm and the categorical cross-entropy loss function. The dropout rate was fixed at 0.3, the batch size at 16, and the validation split rate at 0.1. The maximum epoch count was set to 100, and an early stopping strategy was applied. The training process was conducted with an 80–20 training/test split ratio.

## 4. Application Results

This section presents the experimental results of the proposed hybrid deep learning-based method and compares the effect of wavelet and hybrid features on model performance. The results obtained have been comprehensively analyzed in terms of model accuracy, generalization ability, and feature contribution. The Top-3 and Top-5 wavelet sets were determined by ranking the F1-macro scores of the hybrid model results obtained for each wavelet family. First, LSTM, BiLSTM, GRU, and BiGRU models were trained for each wavelet family; then, the model–wavelet combination with the highest F1 value was selected. As a result of this ranking, the three wavelet families with the highest F1-macro values (coif1, bior3.3, db2) formed the “Top-3” set, while the first five families (coif1, bi-or3.3, db2, bior3.5, rbio3.3) formed the “Top-5” set. This selection process was based not only on proportional improvement but also on absolute performance level (balanced accuracy and F1-macro). Thus, the Top-3 and Top-5 groups were determined in a way that would provide diversity in terms of both model performance and the time-frequency representation of different wavelet families. Table 6 shows the effect of wavelets on classification models.

The gain value indicates the performance increase provided by hybrid features compared to the wavelet-based model alone and is calculated as given in Equation (3). This metric shows the relative contribution of feature fusion to classification accuracy and generalization ability in percentage terms.

(3) $${Gain}_{\left(\%\right)}=\left({Metric}_{Hybrid}-{Metric}_{Wavelet-Only}\right)*100$$

When examining Table 6, although some wavelet families, such as bior3.5, showed a high increase with hybrid feature fusion F1-macro, fluctuations in balanced accuracy values were observed in these models. This situation can lead to the misrepresentation of some classes in data with class imbalance. Therefore, priority was given to combinations that provide consistent and high performance in both F1-macro and balanced accuracy metrics during the selection process. Furthermore, the three selected wavelet families (coif1, bior3.3, and db2) represent different wavelet types (coiflet, biorthogonal, and daubechies), ensuring a balance of diversity in terms of time–frequency resolution. This approach enhances the generalization capability of the hybrid model and highlights the complementary role of wavelet families in echo-based material classification.

In the selection of the top-5 wavelet families, the bior3.3, db2, coif3, coif1, and rbio3.3 families were determined to be the best five-fold combination. These wavelets effectively represent the time–frequency components of echo signals at different scales by offering a balanced distribution of symmetric–asymmetric and orthogonal–biorthogonal structures.

### 4.1. Model Performance

This section presents the classification performance of the proposed method under different model and feature scenarios. In the first stage, LSTM, BiLSTM, GRU, and BiGRU models were trained using only wavelet features. The training was repeated in the Top-3, Top-5, and All-Wavelet Features scenarios, respectively, to analyze the effect of the number of wavelet-based features on model performance. As a result of this analysis, it was observed that the hybrid feature set, which adds acoustic features to the wavelet features, provided an average accuracy increase of 27–29%. Table 7 shows the model training results for the Top-3 wavelet family.

**Figure 4:**
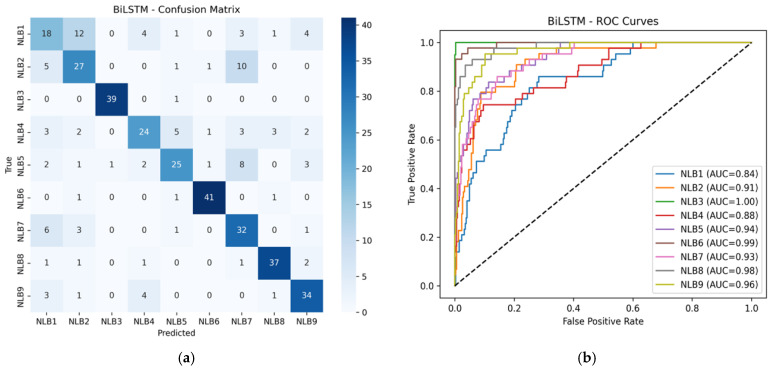

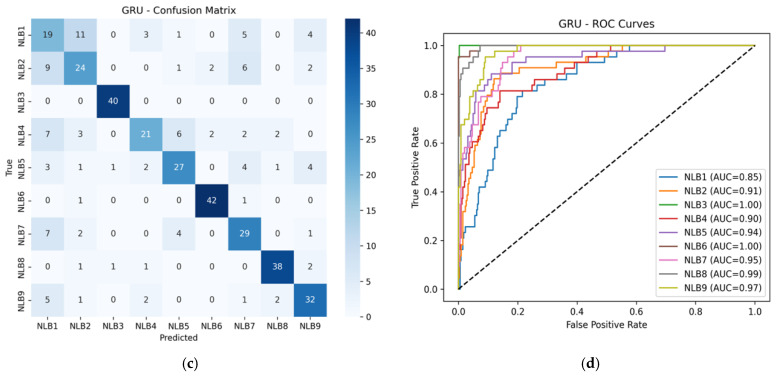
Confusion matrix and ROC-AUC graphs for BiLSTM and GRU models. (**a**) Confusion Matrix for BiLSTM, (**b**) ROC-AUC Curve for BiLSTM, (**c**) Confusion Matrix for GRU, (**d**) ROC-AUC Curve for GRU, (NLB1: “Wood”, NLB2: “White Goods”, NLB3: “Glass”, NLB4: “Iron”, NLB5: “Foam”, NLB6: “Marble”, NLB7: “PVC”, NLB8: “Metal Sheet”, NLB9: “Brick”).

Table 7 shows the comparative performance results of deep learning models trained only with wavelet-based and hybrid feature sets. As seen, adding hybrid features resulted in an increase of approximately 50% in balanced accuracy and F1-macro scores across all models. ROC-AUC values also rose to the 93–94% range in the hybrid structure, revealing a significant improvement in inter-class discrimination power. BiLSTM and GRU-based models were observed to exhibit the highest accuracy and stability with hybrid features. Figure 4 shows the confusion matrices and ROC-AUC scores for the BiLSTM and GRU models.

According to the BiLSTM results in Figure 4a,b, the model was able to distinguish between the Glass (NLB3) and Marble (NLB6) classes with high accuracy. Similarly, the GRU model shown in Figure 4c,d also achieved high AUC values (≥95%) in the Metal Sheet (NLB8) and PVC (NLB7) classes. The fact that the AUC values are above 85% in all classes indicates that the models have a strong overall discrimination ability. However, there are partial mix-ups in the Wood (NLB1) and Brick (NLB9) classes, which is thought to be due to the acoustic echo characteristics of these classes being close to each other. Both materials have a porous structure.

Based on the comparative analysis of the Top-3 wavelet functions presented in Table 7, additional experiments were conducted by extending the evaluation to the Top-5 performing wavelets to further validate the consistency of the obtained results which are given in Table 8.

**Figure 5:**
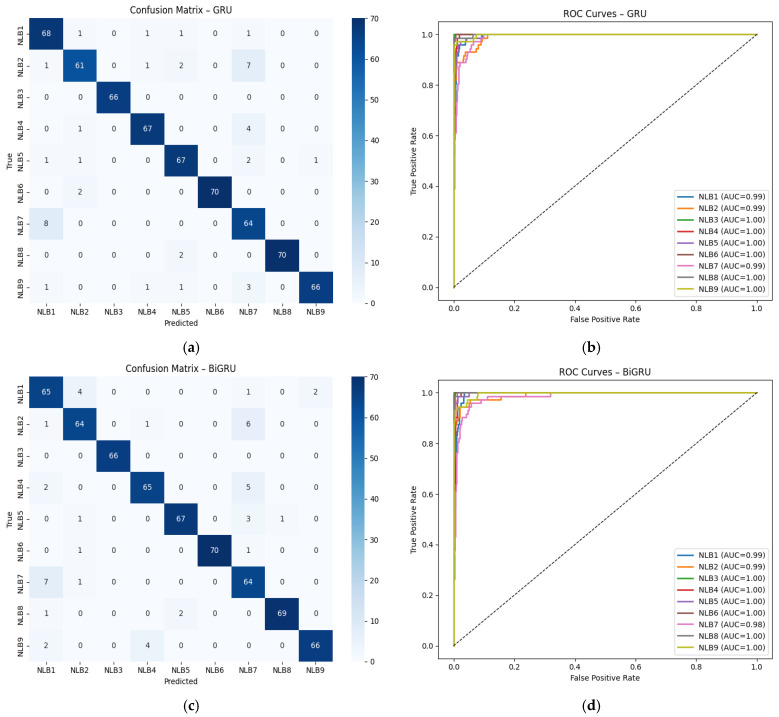
Confusion matrix and ROC-AUC graphs for GRU and BiGRU models. (**a**) Confusion Matrix for GRU, (**b**) ROC-AUC Curve for GRU, (**c**) Confusion Matrix for BiGRU, (**d**) ROC-AUC Curve for BiGRU, (NLB1: “Wood”, NLB2: “White Goods”, NLB3: “Glass”, NLB4: “Iron”, NLB5: “Foam”, NLB6: “Marble”, NLB7: “PVC”, NLB8: “Metal Sheet”, NLB9: “Brick”).

Table 8 shows the performance comparison of models trained using the top five wavelet families (Top-5) in Wavelet-Only and Hybrid scenarios. The results reveal that hybrid feature fusion significantly improves model performance. In all models, balanced accuracy and F1-macro values increased from around 20% to over 90%; ROC-AUC values reached 99%, indicating that classification reliability has reached a near-perfect level. GRU and BiLSTM models demonstrated the highest accuracy and generalization performance with hybrid features. Figure 5 shows the confusion matrices and ROC-AUC scores for the BİGRU and GRU models.

When examining Figure 5a,b, according to the GRU results, the model achieved high accuracy rates across all classes and performed nearly flawless classification, particularly in the Glass (NLB3), Marble (NLB6), and Metal Sheet (NLB8) classes. According to the ROC curves, the AUC values for all classes are in the range of 0.98–1.00. Similarly, the BiGRU Figure 5c,d, also demonstrated similar performance, reaching AUC = 1.00, particularly in the PVC (NLB7), White Goods (NLB2), and Foam (NLB5) classes. The confusion matrices generally reveal that the cross-class confusion is quite low and that the models have high generalization capacity. This situation shows that GRU-based architectures can effectively capture time–frequency relationships after hybrid feature fusion.

The Top-5 analysis provided valuable insights into the most effective wavelet types; however, to obtain a broader understanding of the model behavior across different wavelet structures, a full-scale comparison involving all families was performed, as detailed in Table 9.

**Figure 6:**
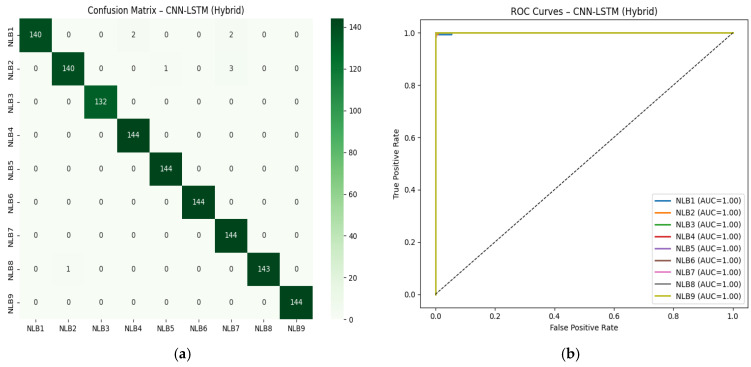
Confusion matrix and ROC-AUC graphs for CNN-LSTM. (**a**) Confusion Matrix for CNN-LSTM, (**b**) ROC-AUC for CNN-LSTM, (NLB1: “Wood”, NLB2: “White Goods”, NLB3: “Glass”, NLB4: “Iron”, NLB5: “Foam”, NLB6: “Marble”, NLB7: “PVC”, NLB8: “Metal Sheet”, NLB9: “Brick”).

Table 9 shows that hybrid feature fusion significantly improves the classification performance of the models. Specifically, LSTM, BiLSTM, GRU, and BiGRU-based models achieved balanced accuracy values of around 25% on average using only wavelet features, while this rate increased to 98% with hybrid features. The fact that ROC-AUC values are at the 99% level for all models indicates that the distinction between classes is achieved with near-perfect accuracy. While the CNN-LSTM architecture achieves the highest F1-macro score with hybrid features, models incorporating attention layers also produce results with high stability, comparable to classical RNN architectures. This table generally shows that hybrid feature fusion significantly strengthens the model’s generalization ability. Figure 6 shows the confusion matrix and ROC-AUC curves for the CNN-LSTM model trained with hybrid features.

As shown in Figure 6a, the model correctly classified all classes with 100% accuracy; no confusion was observed. This demonstrates that the model has a very high capacity to process both temporal and spectral features together. The ROC curves also support this result (see in Figure 6b) with AUC = 1.00 achieved across all classes. This result shows that the CNN layers effectively learned the frequency patterns, while the LSTM layers effectively learned the temporal dependencies of the echo signals, resulting in excellent discrimination performance.

### 4.2. Training Analysis

**Figure 7:**
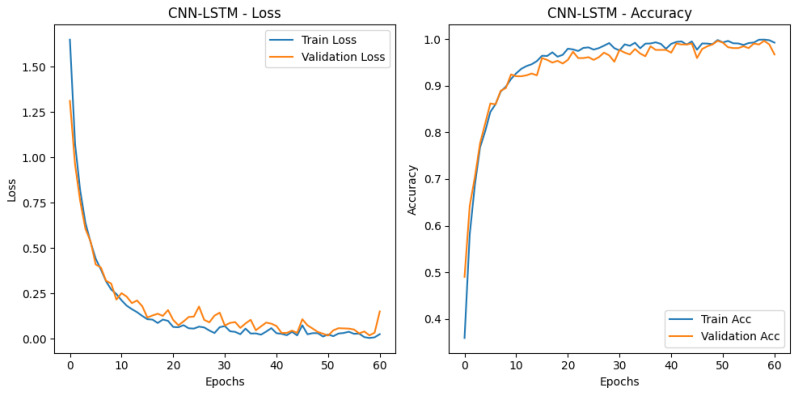
CNN-LSTM training and validation-loss curves graph.

This section presents the analysis results regarding the training process of the proposed CNN–LSTM model. The training and validation loss graph given in Figure 7 clearly shows that the model does not exhibit overfitting tendencies throughout the learning process. Examining the graph, the training and validation losses follow a similar trend, with both curves steadily decreasing as the epoch progresses. This indicates that the model not only memorizes the training data but also possesses high generalization capability. The training process was defined as 60 epochs, but the best performance was achieved around the 35th epoch using the early stopping criterion.

The rapid decrease in the loss value and its stabilization after the 20th epoch prove that the CNN layers can effectively learn frequency-related patterns, while the LSTM layers can effectively learn temporal dependencies. As a result, the CNN-LSTM model not only achieved high accuracy but also demonstrated superior performance compared to other deep learning models in terms of learning stability and generalization capability. In short, compared to other deep learning models, the CNN–LSTM architecture provided lower loss values and faster convergence in both the training and validation phases. Therefore, only the learning process of the CNN–LSTM model is presented in Figure 7, and it is stated that the model exhibits the most stable and optimal training behavior.

### 4.3. Feature Redundancy Analysis

To identify potential redundancies in the feature space, Pearson correlation analysis was applied to the 70-dimensional feature set, and feature pairs showing a high degree of linear relationship were identified using the threshold value $\left|r\right|>0.85$. The analysis revealed that 28 features were strongly correlated with other features, and these features were removed from the dataset. Thus, the number of features was reduced from 70 to 42. Furthermore, L1 regularization was added to the training process to enable the model to learn a sparser representation by suppressing redundant feature weights. L1 regularization reduces the potential overfitting effect by bringing the weights of low-contribution features closer to zero, thereby helping the model develop a more streamlined decision mechanism. The CNN–LSTM model was retrained with the reduced 42-dimensional feature set, and performance evaluation was performed using a 5-fold cross-validation method. The layer-based performance results are presented in Table 10, while the mean and standard deviation values are presented in Table 11.

Table 10 shows that the Balanced Accuracy and Macro F1-Score values for each class are high and consistent.

Table 11 reveals that the model operates at a general accuracy level of 98% and demonstrates stable generalization performance thanks to low standard deviation.

These findings show that removing highly correlated features and applying L1 regularization does not cause a significant loss in model performance. In other words, the results obtained with the reduced feature set confirm that the model is structurally robust and resilient to outliers. However, since the primary objective of the study was to comprehensively examine the relationships between material types and acoustic and wavelet-based features, analyses using the full feature set were also evaluated, preserving interpretability and physical meaningfulness.

### 4.4. Feature Importance Analysis

**Figure 8:**
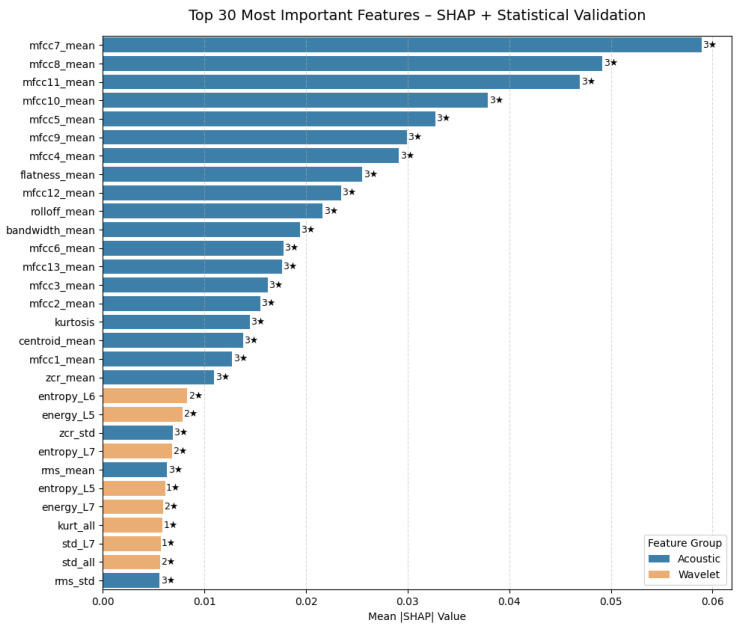
CNN-LSTM Top-30 feature (SHAP analysis) (“⋆” represents statistical selection power).

In this section, the features that influence the decision-making process of the CNN-LSTM (hybrid) model were examined using explainable artificial intelligence (XAI)-based methods. Feature importance levels were first calculated using the SHAP method. Additionally, statistical methods such as ANOVA F-score, mutual information, and RFE were used solely for robustness validation. This ensured that the features prominent in the model’s decision mechanism were evaluated in terms of both explainability and statistical consistency. Figure 8 shows the contribution level of the top 30 features with the highest average SHAP value according to the CNN-LSTM model to the model predictions.

In the graph in Figure 8, most of the features with the highest impact are MFCC-based parameters belonging to the acoustic group. Specifically, the mfcc7_mean, mfcc8_mean, and mfcc11_mean features are the components that contribute most to the model’s decision mechanism in distinguishing mate-rial classes. This indicates that cepstral coefficients in the mid-frequency band of echo signals play a critical role in determining material type. Among the wavelet-based features, entropy_L6, energy_L5, and energy_L7 stand out. These features represent the energy density and irregularity level of the signal in the time-frequency domain and, together with the acoustic features, provide a complementary contribution to the overall performance of the model. In conclusion, SHAP analysis confirms that acoustic and wavelet-based features work complementarily in a hybrid structure. While coefficients belonging to the MFCC family play a dominant role in the model’s predictive power, wavelet entropy and energy components are auxiliary features that clarify class boundaries. The asterisks (⋆) in the graph indicate statistical selection power:

- 1⋆: Feature selected as significant by only one method (e.g., ANOVA, MI, RFE).
- 2⋆: Feature consistently selected by two methods.
- 3⋆: Feature found significant in all methods (ANOVA, MI, RFE) and with high robustness.

**Figure 9:**
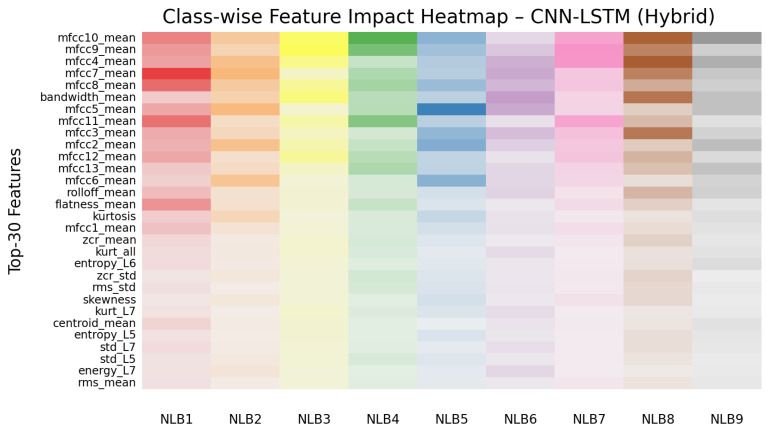
CNN-LSTM class-based SHAP analysis (Darker colors represent dominant features, while lighter colors represent less dominant features).

Therefore, the features marked with 3 at the top of the graph are considered the most reliable attributes in terms of both SHAP explainability analysis and statistical validation methods. The class-based SHAP analysis is shown in Figure 9.

The features that the model emphasizes most for wood (NLB1) surfaces are determined as mfcc7_mean, mfcc9_mean, mfcc11_mean, mfcc, and flatness_mean. These components reflect the complex resonance structure of wood in the mid-to-high frequency ranges [22]. The effect of high-order MFCCs is consistent with the tonal diversity and bright sound components that arise from the natural fiber structure of wood. Furthermore, the bandwidth_mean and flatness_mean values indicate that wood distributes its sound energy across very different frequencies, i.e., it has a complex surface with a broad spectral distribution [28]. Therefore, the model defines wood materials based on their natural resonance and mid-to-high frequency richness.

The most influential features found in the decision process of the CNN–LSTM model in the white goods (NLB2) class were mfcc7_mean, mfcc2_mean, mfcc6_mean, mfcc5_mean, mfcc4_mean, and kurtosis. These features represent the high-frequency [8] reflection behavior resulting from white goods typically having metal coatings or hard plastic surfaces. The medium and low-order MFCCs (mfcc2_mean–mfcc7_mean range) represent the energy in both the fundamental and mid-frequency regions of the signal; this enables the model to recognize short-term echoes formed depending on the hardness of the surface. The high kurtosis value indicates that the signal energy is concentrated at sudden peaks in time [19], meaning that the sound reflects brightly and sharply from the surfaces of white goods. Therefore, the model distinguishes the white goods class by a combination of high peak energy, mid-frequency dominance, and short-duration metallic echoes.

For glass (NLB3) surfaces, the features that stood out in the SHAP analysis were mfcc9_mean, bandwidth_mean, mfcc10_mean, mfcc12_mean, mfcc11_mean, and mfcc8_mean. These features represent the high-frequency energy [17] accumulation caused by the glass’s high hardness coefficient and fully reflective surface properties. In particular, the high-order cepstral coefficients in the mfcc8_mean–mfcc12_mean range indicate that glass reflects sound energy at high frequencies and in a narrow-band manner. The relatively high bandwidth_mean value indicates that the sound spectrum is spread over a wide frequency range, but that most of the energy is concentrated in the upper harmonics. This is entirely consistent with glass’s typical behavior of producing a bright, sharp, and non-resonant echo. Consequently, the model characterizes glass through high-frequency reflections, a broad-band spectral distribution, and high-order MFCC contributions.

The prominent features for iron (NLB4) surfaces are defined as mfcc10_mean, mfcc11_mean, mfcc9_mean, flatness_mean, std_L5, zcr_std, and rms_std. These parameters represent the tendency of iron, due to its high density and hardness, to reflect sound energy in the form of strong, short-duration, and wide-band echoes [19]. High-order MFCCs (mfcc9–mfcc11) reveal high-frequency resonances on iron surfaces, while the flatness_mean value indicates that these ech-oes have a complex and scattered spectral structure. The high values of rms_std and zcr_std, which represent variations in the time domain, characterize sudden amplitude changes and rapid transitions in the signal. Consequently, the model distinguishes iron surfaces based on high-frequency energy, sudden reflection transitions, and complex spectral distribution.

The features that stand out in the foam (NLB5) class are mfcc5_mean, mfcc2_mean, mfcc6_mean, kurtosis, skewness, and entropy_L5. Since foam is an acoustically highly absorbent material [17], the energy of the signal reflected from its surface is quite low and its frequency structure is irregular. The coefficients in the mfcc2–mfcc6 range represent weak but dispersed energy components in the low and mid-frequency regions. The high entropy_L5 value indicates a random energy distribution due to the reverberation-free nature of foam, while the kurtosis and skewness values show that the signal has a low amplitude but statistically unbalanced structure. The model therefore defines foam as having high entropy, low energy density, and a low-frequency-focused spectral structure.

In the marble (NLB6) class, the features that contributed most to the model’s decision process were energy_L7, std_L7, kurt_L7, kurt_all, bandwidth_mean, mfcc4_mean, mfcc7_mean, mfcc5_mean, and mfcc3_mean. The dense and uniform [15] surface structure of marble causes the echo character to have a short pulse shape with high energy. The energy_L7 and kurt_L7 features represent the high peak energy of these dense reflections. The high value of bandwidth_mean indicates that the reflected sound is spread over a wide frequency range, while the coefficients in the mfcc3–mfcc7 range explain the resonance richness in the mid-frequency region. Therefore, the model distinguishes marble surfaces through high-energy, short-duration echoes and a wide-band spectral response.

The features that stand out in the SHAP analysis for PVC (NLB7) surfaces are mfcc9_mean, mfcc4_mean, mfcc10_mean, mfcc11_mean, mfcc2_mean, skewness, flatness_mean, and rms_mean. Since PVC is a semi-rigid and partially flexible polymer [17], the sound reflected from its surface has both a reflected and partially absorbed character. The coefficients in the mfcc2–mfcc11 range clearly show this mid–high frequency balance. The moderate levels of flatness_mean and skewness indicate that the signal has a regular but frequency-scattered structure. Furthermore, the distinct rms_mean value indicates that the echo energy of PVC surfaces is distributed homogeneously. These findings reveal that the model recognizes PVC through its mid-frequency resonances, balanced energy distribution, and semi-absorbent surface effect.

The features found to be effective in the metal sheet (NLB8) class are determined as mfcc4_mean, mfcc10_mean, mfcc3_mean, bandwidth_mean, rolloff_mean, entropy_L5, std_L7, zcr_mean, and zcr_std. These parameters clearly reflect the broadband and high-frequency echo profile [8] resulting from the thin, flexible, and fully reflective nature of the metal sheet. The high values of rolloff_mean and bandwidth_mean indicate that energy is concentrated in the upper region of the spectrum, meaning the surface creates a metallic brightness effect. The values of zcr_mean and zcr_std indicate that the signal frequently crosses zero, which means high-frequency, thin surface reflections. Therefore, the model characterizes metal sheet surfaces through high-frequency dominance, broadband reflections, and high temporal variability.

The features that stand out in the SHAP analysis for the brick (NLB9) class are mfcc10_mean, mfcc4_mean, mfcc2_mean, mfcc13_mean, entropy_L6, and centroid_mean. Brick, with its porous and rough surface structure [17], absorbs most of the sound energy and brings low-frequency components to the fore. The low-order cepstral coefficients mfcc2_mean and mfcc4_mean represent the effect of this low-frequency energy, while higher-order coefficients such as mfcc10 and mfcc13 capture the micro-resonance diversity caused by surface roughness. The high entropy_L6 value indicates that the reflected signal has an irregular and dispersed energy structure [22]. Furthermore, the low cen-troid_mean value confirms that the brick’s echo is concentrated in the lower frequencies. These findings show that the model recognizes the brick through its low-frequency dominance, high surface entropy, and roughness-induced resonance components.

## 5. Discussion

In this study, the echo-based material recognition problem was analyzed on the ANLOS-R dataset created under NLOS conditions. NLOS conditions are environments where sound cannot reach the target surface directly, and information can only be obtained indirectly through reflection and refraction paths. Therefore, material classification in this environment requires models that can analyze the time, frequency, and energy content of the echo in a multidimensional manner. In this context, three different feature levels were created within the scope of the study:

- Hybrid acoustic features (MFCC1–13, RMS, ZCR, etc.) represented the classical spectral energy in the time-frequency domain.
- Wavelet features were extracted using ten different wavelet families (bior3.3, bior3.5, coif1, coif3, db2, db4, db6, rbio3.3, sym2, sym5) and captured the multi-scale energy and entropy distribution of the signal.
- The fusion (Hybrid + Wavelet) set combined these two information sources in a Z-score normalized joint space.

Unlike classical acoustic features, the wavelet transform could simultaneously analyze both the temporal and frequency-related changes in the signal but did not provide sufficient discrimination. At the same time, classical acoustic features alone are not sufficient for material classification in echo signals. For this reason, feature fusion (Hybrid + Wavelet) was used. This allowed modeling multiple echo components arising in the NLOS environment, depending on the length of the echo paths and the surface properties of the material.

Eight different deep learning-based time series models were tested in the study: LSTM, BiLSTM, GRU, BiGRU, GRU–Attention, BiGRU–Attention, BiLSTM–Attention, and CNN–LSTM. These models were trained with both wavelet-only and fusion (hybrid) features. The results show that the Wavelet–Hybrid feature set provided significantly higher performance than the Wavelet-Only set in all models. While the balanced accuracy value of the Wavelet-Only models remained at around 25%, this value reached the range of 97–99% in the Wavelet–Hybrid models. Similarly, the F1-macro and ROC–AUC metrics also reached near-perfect classification levels in the hybrid representation. The highest accuracy rate was achieved by the CNN–LSTM architecture (balanced accuracy: 0.99, F1-macro: 0.99, ROC–AUC: 0.99). The CNN–LSTM structure successfully analyzed the complex nature of echoes by combining the spectral pattern extraction power of CNN layers with the temporal dependency modeling capability of LSTM layers. While CNN learned the frequency-based distribution of the echo, LSTM modeled the time-dependent evolution of these frequency components. This allowed the system to represent both reflected and absorbed energy components simultaneously.

The model’s decision processes were analyzed using the SHAP method. Class-based SHAP maps showed that the model distinguished material types based on physically meaningful acoustic parameters. For wood (NLB1), the mfcc7_mean, mfcc8_mean, mfcc11_mean, and flatness_mean features were dominant. This is consistent with wood’s tendency to strongly reflect energy in the mid-frequency spectrum. For glass (NLB3), the mfcc9_mean, bandwidth_mean, and mfcc10–12 coef-ficients were decisive, supported by glass’s high-frequency bright echo production. For marble (NLB6), the prominence of wavelet-based statistics such as energy_L7, std_L7, and kurt_L7 indicated the nature of hard surfaces to produce short-duration, strong echoes. In the Foam and Brick (NLB5, NLB9) classes, high entropy_L5–L6 values reflected the irregular distribution of energy due to the porous structure. The high importance of wavelet levels (especially L5–L7) in the SHAP analysis revealed that material-discriminating information is concentrated in the mid-low frequency bands under NLOS echo conditions. These bands are directly related to physical parameters such as echo du-ration, surface hardness, density, and porosity.

The results prove that the Wavelet–Hybrid CNN–LSTM architecture, which uses both wavelet and acoustic features, exhibits high generalizability even in NLOS environments. This model not only offers a high accuracy rate but also presents a physically explainable decision mechanism. Therefore, the model establishes a strong reference in the field of acoustic NLOS-based material recognition at both theoretical and applied levels.

## 6. Conclusions and Future Work

In this study, the material recognition problem from echo signals was investigated using the ANLOS-R dataset, and high-accuracy classification performance was achieved with the Wavelet–Hybrid CNN–LSTM architecture. Compared to classical MFCC-based approaches, incorporating multi-scale energy and entropy statistics derived from wavelet transformation into the model better represented the physical nature of the echo, particularly under NLOS conditions, and significantly improved performance. The SHAP-based interpretability analysis of the model showed that the learned features are directly related to the physical properties of the material (hardness, density, porosity, surface roughness).

This finding demonstrates that deep learning models can be evaluated not only as classification tools but also as systems that can interpret acoustic material physics. In future work, (i) transfer learning-based approaches can be applied to generalize the model to different acoustic environments, (ii) lightweight CNN-LSTM architectures can be developed for use in the real-time applications, and (iii) the model’s behavior can be validated with 3D acoustic simulation environments to create a broader application framework in echo-based acoustic perception systems.

Additionally, as part of future work, it is planned to add synthetic noise to increase the acoustic diversity of the dataset and to include different reflective surface materials (glass, wood, metal, etc.) in the experimental environment. This approach will allow for the evaluation of the generalizability of the proposed method to different reverberation characteristics and complex acoustic environments.

The proposed hybrid feature representations, based on the physical characteristics of the echo signal, have the potential to be adapted to different NLOS acoustic sensing tasks through transfer learning. Future work will experimentally evaluate the reusability of the learned intermediate layer representations across different tasks.

In conclusion, this study demonstrates that the Wavelet–Hybrid CNN–LSTM model is an innovative approach that offers both high accuracy and interpretability in NLOS echo conditions.

## Figures and Tables

**Table 1:** Comparative summary of acoustic NLOS studies and contribution of the proposed study.

Reference	Method	Objective	Contribution
[14]	CAM + LSTM-based 1D-CNN	Automatic classification of NLOS acoustic signals	Important features were highlighted using the CAM, temporal dependencies were modeled using LSTM; 98% accuracy was achieved.
[15]	Wavelet transform optimized with genetic algorithm	Resonance spectrum analysis and acoustic signal noise reduction	Wavelet scales were selected using a genetic algorithm to remove noise, reducing regression and classification errors by 95% and 40%, respectively.
[16]	Hybrid feature extraction	Classification of acoustic signals	Deep learning-based features were combined with statistical features, achieving 77.66% classification success with linear SVM.
[17]	Image-based material classification + RIR estimation	Echo-based acoustic material modeling	Automatic echo response generation was achieved by estimating acoustic absorption coefficients from visual surface patches.
[18]	CWT + CNN/CAE	Pipeline leak detection	CWT-based acoustic images were processed with CNN and an auto-encoder, and high accuracy was achieved by extracting both global and local features.
[19]	CNN-based automatic feature extraction (FFT + t-SNE/PCA)	Acoustic emission signal analysis	High accuracy was achieved in wear and fault detection using CNN-based automatic feature extraction.
[20]	Multi-scale CNN + CAM	Bearing fault detection in noisy environments	Channel-based weighting was applied with CAM, achieving high accuracy and low processing time in anti-noise experiments.
**This study**	**Wavelet-acoustic feature fusion + LSTM/GRU-based deep networks + CNN-LSTM + SHAP analysis**	**NLOS echo-based material classification**	**Material recognition from echo signals by combining different wavelet and acoustic features; feature importance ranking was performed with SHAP-based interpretability.**

**Table 2:** General information about ANLOS-R dataset.

File Name	Object Type	Position 0	Location 1 (+5 cm)	Location 2 (−5 cm)
NLB0	Empty	NLB0-0	NLB0-1	NLB0-2
NLB1	Wood	NLB1-0	NLB1-1	NLB1-2
NLB2	White Good	NLB2-0	NLB2-1	NLB2-2
NLB3	Glass	NLB3-0	NLB3-1	NLB3-2
NLB4	Iron	NLB4-0	NLB4-1	NLB4-2
NLB5	Foam	NLB5-0	NLB5-1	NLB5-2
NLB6	Marble	NLB6-0	NLB6-1	NLB6-2
NLB7	PVC	NLB7-0	NLB7-1	NLB7-2
NLB8	Metal Sheet	NLB8-0	NLB8-1	NLB8-2
NLB9	Brick	NLB9-0	NLB9-1	NLB9-2

**Table 3:** Calculated basic statistical features.

Statistical Features	Description
Energy (E)	Represents the total power content of the signal at a given wavelet level.
Entropy (H)	Measures the degree of disorder and energy spread of the echo signal.
Mean (μ)	Indicates the general trend of coefficients at each wavelet level and represents the average energy level of the signal for that scale.
$\mathrm{Standard}\;\mathrm{deviation}\;\left(\sigma \right)$	Measures the spread of coefficients around the mean; reflects the amount of variation in the frequency band.
Skewness	Indicates the degree of symmetry in the coefficient distribution; positive or negative skewness indicates an asymmetric concentration of echo energy.
Kurtosis	Measures the peak intensity of the distribution; high kurtosis indicates the presence of sharp and energy-dense components in the echo signal.

**Table 4:** Structure of hybrid and wavelet-based feature set.

Feature Type	Source	Dimension	Description
Acoustic	RMS, ZCR, MFCC1-13	24	Time domain + spectral summaries
Wavelet	bior3.3, bior3.5, coif1., coif3, db2, db4, db6, rbio3.3, sym2, and sym5	46	Multiscale energy and entropy statistics
**Fusion**	**Acoustic + wavelet**	**70**	**Z-score normalized and combined feature set**

**Table 5:** Architectural structure and training parameters of deep learning models.

Model	Architecture	Hidden Units	Layers	Epochs	Attention
LSTM	LSTM (128) Dense (64, ReLU) Softmax	128	3	60 (max) 35 (e.s. ^1^)	-
BiLSTM	BiLSTM (128) Dense (64, ReLU) Softmax	128 × 2	3	60 (max) 37 (e.s.)	-
GRU	GRU (128) Dense (64, ReLU) Softmax	128	3	60 (max) 32 (e.s.)	-
BiGRU	BiGRU (128) Dense (64, ReLU) Softmax	128 × 2	3	60 (max) 34 (e.s.)	-
GRU-Attention	GRU (128) Attention Dense (64, ReLU) Softmax	128	4	60 (max) 36 (e.s.)	Temporal
BiGRU-Attention	BiGRU (128) Attention Dense (64, ReLU) Softmax	128 × 2	4	60 (max) 39 (e.s.)	Bidirectional
BiLSTM-Attention	BiLSTM (128) Attention Dense (64, ReLU) Softmax	128 × 2	4	60 (max) 40 (e.s.)	Bidirectional
CNN-LSTM	CNN1D (64, k = 3, ReLU) LSMT (128) Dense (64, ReLU) Softmax	64 + 128	4	60 (max) 35 (e.s.)	Bidirectional

^1^ early stop.

**Table 6:** Effect of wavelets on each classification model.

Wavelet	Model	Wavelet-Only		Hybrid		Gain Score	
		Balanced Accuracy	F1-Macro	Balanced Accuracy	F1-Macro	Gain Accuracy	Gain F1-Macro
bior3.3	GRU	0.19	0.19	0.56	0.56	36.87%	37.34%
bior3.5	GRU	0.15	0.11	0.55	0.55	39.52%	44.46%
coif1	BiGRU	0.22	0.20	0.58	0.57	35.44%	37.75%
coif3	BiLSTM	0.17	0.14	0.51	0.52	34.11%	37.28%
db2	GRU	0.19	0.16	0.56	0.56	37.87%	40.52%
db4	GRU	0.18	0.17	0.52	0.52	33.85%	35.21%
db6	BiLSTM	0.19	0.15	0.52	0.52	33.63%	37.27%
rbio3.3	BiGRU	0.13	0.12	0.52	0.51	38.61%	39.59%
sym2	BiLSTM	0.16	0.10	0.50	0.51	34.75%	40.40%
sym5	GRU	0.16	0.13	0.56	0.55	39.94	42.02

**Table 7:** Top-3 wavelet family classification results.

Model	Wavelet-Only			Hybrid		
	Balanced Accuracy	F1-Macro	ROC-AUC	Balanced Accuracy	F1-Macro	ROC-AUC
LSTM	0.21	0.17	0.66	0.70	0.70	0.93
BiLSTM	0.23	0.18	0.64	0.71	0.71	0.93
GRU	0.22	0.18	0.66	0.70	0.70	0.94
BiGRU	0.22	0.20	0.65	0.69	0.69	0.94

**Table 8:** Top-5 wavelet family classification results.

Model	Wavelet-Only			Hybrid		
	Balanced Accuracy	F1-Macro	ROC-AUC	Balanced Accuracy	F1-Macro	ROC-AUC
LSTM	0.23	0.22	0.68	0.91	0.91	0.99
BiLSTM	0.22	0.21	0.69	0.91	0.91	0.99
GRU	0.22	0.21	0.68	0.93	0.93	0.99
BiGRU	0.21	0.20	0.68	0.92	0.93	0.99

**Table 9:** All wavelet family and all wavelet-acoustic classification results.

Model	Wavelet-Only			Hybrid		
	Balanced Accuracy	F1-Macro	ROC-AUC	Balanced Accuracy	F1-Macro	ROC-AUC
LSTM	0.28	0.27	0.72	0.98	0.98	0.99
BiLSTM	0.26	0.24	0.72	0.98	0.98	0.99
GRU	0.25	0.23	0.70	0.98	0.98	0.99
BiGRU	0.24	0.23	0.69	0.98	0.98	0.99
GRU-Attention	0.26	0.24	0.71	0.97	0.97	0.99
BiGRU-Attention	0.25	0.23	0.70	0.98	0.98	0.99
BiLSTM-Attention	0.25	0.25	0.70	0.98	0.98	0.99
CNN-LSTM	0.25	0.23	0.70	0.99	0.99	0.99

**Table 10:** Five-fold cross-validation results of the CNN–LSTM model with 42 features.

Fold	Balanced Accuracy	Macro F1-Score
Fold 1	0.99	0.99
Fold 2	0.99	0.99
Fold 3	0.97	0.97
Fold 4	0.99	0.99
Fold 5	0.97	0.97

**Table 11:** Average performance and standard deviation values obtained from 42 features.

Metric	Mean	Standard Deviation (±)
Balanced Accuracy	0.98	0.0085
Macro F1-Score	0.98	0.0085

## Data Availability

The data presented in this study will be available on request from the corresponding author.

## Abbreviations

The following abbreviations are used in this manuscript:

LOS	Line of Sight
NLOS	Non-Line of Sight
ANLOS-R	Acoustic Non-Line of Sight- Reflection
LSTM	Long Short-Term Memory
BİLSTM	Bidirectional Long Short-Term Memory
GRU	Gated Recurrent Unit
CNN-LSTM	Convolutional Neural Network—Long Short-Term Memory
SHAP	SHapley Additive exPlanations
MFCC	Mel-Frequency Cepstral Coefficients
UWB	Ultra-Wideband
SNR	Signal-to-Noise Ratio
RMS	Root Mean Square
ZCR	Zero Crossing Rate
SYM	Symlet
BİOR	Biorthogonal
COİF	Coiflet
DB	Daubechies
RBİO	Reverse Biorthogonal
ROC	Receiver Operating Characteristic
AUC	Area Under the Curve
MIMO	Multiple-Input Multiple-Output
SC	Spectral Centroid
SB	Spectral Bandwidth
SR	Spectral Rolloff
SF	Spectral Flatness
1D-CNN	One-Dimensional Convolutional Neural Network
CAM-LSTM	Class Activation Map—LSTM
CAM	Class Activation Map
NASNetLarge	Neural Architecture Search Network (Large)
NASNetMobile	Neural Architecture Search Network (Mobile)
SVM	Support Vector Machine
RIR	Room Impulse Response
CWT	Continuous Wavelet Transform
CAE	Convolutional Autoencoder
FFT	Fast Fourier Transform
T-SNE	t-distributed Stochastic Neighbor Embedding
PCA	Principal Component Analysis

## References

[1] Aicha Y., Med S.C., Chellil O.A. (2011). Identification of defects in composite materials using an improved Wavelet analysis algorithm. Phys. Int..

[2] Yury M., Yury S., Olga L., Alexandra S. (2020). Method of analysis and classification of acoustic emission signals to identify Pre-Seismic anomalies. Adv. Sci. Technol. Eng. Syst. J..

[3] Li S., Wu J., Liu X. NLoS recognition and feature importance analysis based on GAN-SSA-RF. Proceedings of the 3rd International Conference on Signal Processing, Computer Networks and Communications.

[4] Kunicki M., Koziol M., Urbaniec I., Zygarlicki J. Wavelet selection for analysis of acoustic signals emitted by partial discharges in oil insulation. Proceedings of the 24th International Scientific Conference on Electric Power Engineering.

[5] Wang H.L., Yang W., Zhan W.D., Jun Y. Feature extraction of acoustic signal based on wavelet analysis. Proceedings of the International Conference on Embedded Software and Systems Symposia.

[6] Dgang S., Kai Y., Haifeng T. Application of wavelet analysis in acoustic signal processing. Proceedings of the 8th International Conference on Electronic Measurement and Instruments.

[7] Wang L., Lei B. Time-frequency analysis of underwater acoustic signal based on improved harmonic wavelet. Proceedings of the IEEE Region 10 International Conference.

[8] Xiong S., Yang N., Guan H., Shi G., Luo M., Deguchi Y., Cui M. (2024). Combination of plasma acoustic emission signal and laser-induced breakdown spectroscopy for accurate classification of steel. Anal. Chim. Acta.

[9] Gao T., Xie Y., Wang X., Qiu L. An improved wavelet denoising method based on acoustic echo analysis. Proceedings of the IEEE International Conference on Control, Electronics and Computer Technology.

[10] Solís M., Benítez-Pérez H., Rubio E., Medina-Gómez L., Moreno E., Gonzalez G., Leija L. (2008). Pattern classification of decomposed wavelet information using ART2 networks for echoes analysis. J. Appl. Res. Technol..

[11] Yao W., Lv D., Zi J., Huang X., Zhang Y., Liu J. Crane song recognition based on the features fusion of GMM based on wavelet spectrum and MFCC. Proceedings of the 7th International Conference on Computer and Communications.

[12] Amal R.J., Kumar C.S., Jose J.A. A wavelet based time-frequency descriptor for automatic classification of acoustic signals of fishes. Proceedings of the 2nd International Conference on Intelligent Computing, Instrumentation and Control Technologies.

[13] Chin C.S., Kek X.Y., Chan T.K. Wavelet scattering based gated recurrent units for binaural acoustic scenes classification. Proceedings of the International Conference on Internet of Things and Intelligent Applications.

[14] Xu J., Liu Y., Jia Y., Guo G., Cao S. (2024). Acoustic NLOS signal recognition based on 1-D convolutional neural network with channel attention mechanism. IEEE Trans. Instrum. Meas..

[15] Greenhall J., Sinha D.N., Pantea C. (2023). Genetic algorithm-wavelet transform feature extraction for data-driven acoustic resonance spectroscopy. IEEE Trans. Ultrason. Ferroelectr. Freq. Control.

[16] Yaman O., Tuncer T. (2021). Ensemble NASNET deep feature generator based underwater acoustic classification model. Veri Bilim..

[17] Colombo M., Dolhasz A., Hockman J., Harvey C. Acoustic rendering based on geometry reduction and acoustic material classification. Proceedings of the IEEE Symposium on Computational Intelligence and Games.

[18] Ahmad S., Ahmad Z., Kim C.H., Kim J.M. (2022). A method for pipeline leak detection based on acoustic imaging and deep learning. Sensors.

[19] González D., Alvarez J., Sánchez J.A., Godino L., Pombo I. (2022). Deep learning-based feature extraction of acoustic emission signals for monitoring wear of grinding wheels. Sensors.

[20] Luo Y., Lu W., Kang S., Tian X., Kang X., Sun F. (2023). Enhanced feature Extraction network based on acoustic signal feature learning for bearing fault diagnosis. Sensors.

[21] Nguyen P.K., Huynh Q.L. (2025). A machine learning approach in EEG-based assessment of cognitive load by mental arithmetic tasks. J. Phys. Conf. Ser..

[22] Xu F., Liu Y., Wang X., Brashaw B.K., Yeary L.A., Ross R.J. (2020). Evaluating internal condition of hardwood logs based on AR-minimum entropy deconvolution combined with wavelet based spectral kurtosis approach. Holzforschung.

[23] Jain A., Verma A., Verma A.K. (2023). Non-invasive and automatic identification of diabetes using ECG signals. Int. J. Electr. Electron. Res..

[24] Atahan Y., Elbir A., Keskin A.E., Kiraz O., Kirval B., Aydin N. Music genre classification using acoustic features and autoencoders. Proceedings of the Innovations in Intelligent Systems and Applications Conference.

[25] Vodnala N., Lankireddy P.R., Yarlagadda P. (2023). Characterization of cough sounds using statistical analysis. arXiv.

[26] Warule P., Chandratre S., Mishra S.P., Deb S. (2024). Detection of the common cold from speech signals using transformer model and spectral features. Biomed. Signal Process. Control.

[27] Tenorio R., Gerosa D. (2025). Scalable data-analysis framework for long-duration gravitational waves from compact binaries using short Fourier transforms. Phys. Rev. D.

[28] Bi F., Yang L. (2025). Research on acoustic scene classification based on time–frequency–wavelet fusion network. Sensors.

[29] Akkaya S. (2025). Wavelet-based denoising strategies for non-stationary signals in electrical power systems: An optimization perspective. Electronics.

[30] Arts L.P.A., Van Den Broek E.L. (2022). The fast continuous wavelet transformation (fCWT) for real-time, high-quality, noise-resistant time–frequency analysis. Nat. Comput. Sci..

